# Cholera

**Published:** 1851-07

**Authors:** 


### NEW POSTAGE LAW.

Of course from this date our correspondents, as everybody else, will pre-pay the postage on all their letters. Our subscribers will have quite a reduction in the postage on the Journal, by pre-paying quarterly in advance. The rates will be as follows:

| | | | |
|---|---|---|---|
| For all distances | under 500 miles, | 1-2 ct. | per oz. |
| " " | over 500 and under 1500 miles, | 1 ct. | " |
| " " | over 1500 " 2500 " | 1 1-2 cts. | " |
| " " | over 2500 " 3500 " | 2 cts. | " |
| " " | over 3500 | 2 1-2 cts. | " |

If the postage be not pre-paid quarterly at the office where the Journal is delivered, these rates will be doubled.

